# Fabrication and Assembly Techniques for Sub-mm Battery-Free Epicortical Implants

**DOI:** 10.3390/mi14020476

**Published:** 2023-02-18

**Authors:** Adam Khalifa, Mehdi Nasrollahpour, Ali Nezaratizadeh, Xiao Sha, Milutin Stanaćević, Nian X. Sun, Sydney S. Cash

**Affiliations:** 1 Department of Electrical and Computer Engineering, University of Florida, Gainesville, FL 32611, USA; 2 Department of Electrical and Computer Engineering, Northeastern University, Boston, MA 02115, USA; 3 Department of Electrical and Computer Engineering, Stony Brook University, Stony Brook, NY 11794, USA; 4 Department of Neurology, Massachusetts General Hospital, Harvard Medical School, Boston, MA 02114, USA

**Keywords:** wireless power transmission, neural stimulation, distributed

## Abstract

Over the past three decades, we have seen significant advances in the field of wireless implantable medical devices (IMDs) that can interact with the nervous system. To further improve the stability, safety, and distribution of these interfaces, a new class of implantable devices is being developed: single-channel, sub-mm scale, and wireless microelectronic devices. In this research, we describe a new and simple technique for fabricating and assembling a sub-mm, wirelessly powered stimulating implant. The implant consists of an ASIC measuring 900 × 450 × 80 µm^3^, two PEDOT-coated microelectrodes, an SMD inductor, and a SU-8 coating. The microelectrodes and SMD are directly mounted onto the ASIC. The ultra-small device is powered using electromagnetic (EM) waves in the near-field using a two-coil inductive link and demonstrates a maximum achievable power transfer efficiency (PTE) of 0.17% in the air with a coil separation of 0.5 cm. In vivo experiments conducted on an anesthetized rat verified the efficiency of stimulation.

## 1. Introduction 

**Figure 1:**
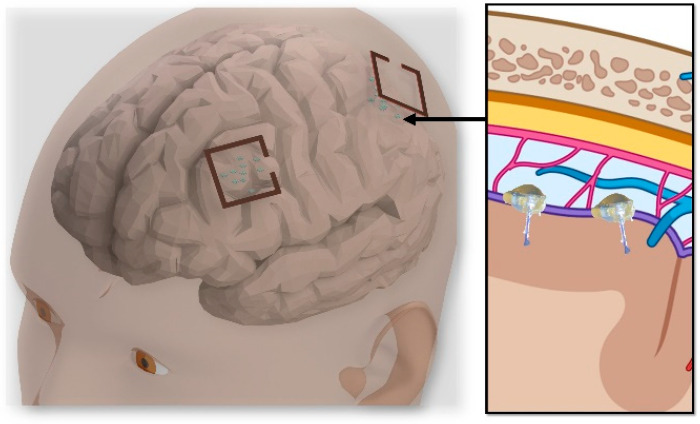
A conceptual diagram of the distributed interface using epicortical, single-channel, wireless implants.

The ongoing need for distributed neural therapies and brain–machine interfaces (BMIs) has motivated researchers to find innovative ways to aggressively miniaturize wireless implants down to the millimeter and sub-millimeter scales. One approach to achieve this is to decentralize the IMD system into several microelectronic devices that are each freely floating, as shown in Figure 1. Examples of recent implementations include the Neurograin [1], the FF-WINeR [2], the MOTE [3], the StimDust [4], and the Microbead [5].

The concept behind distributed interfaces is to increase the coverage of brain areas at the expense of density. For example, single-channel implants can be distributed across multiple regions in the brain and reach areas inaccessible by conventional IMDs (such as folded cortices), giving clinicians and researchers greater specificity in their BMI experiments or close-loop stimulation therapies. Additionally, miniaturized wireless implants would lead to less invasive surgery and shorter hospital stays, as well as a reduced risk of infection.

The distributed approach, however, also brings new challenges and questions, such as how to efficiently transmit wireless power to such small devices and how to miniaturize the application-specific integrated circuit (ASIC) and its packaging. Miniaturized implants are often built around CMOS ASICs due to their ability to be extremely scaled down and their low power consumption [6]. However, one major disadvantage of ASICs is their high microfabrication costs. Unfortunately, there are no viable alternatives to CMOS, as other technologies (e.g., off-the-shelf ICs and surface mount components on PCB) result in bulkier implants. Once the ASIC is fabricated by a semiconductor manufacturer, it must undergo a CMOS-compatible process flow to complete the wireless neural implant. Unfortunately, post-CMOS processing typically involves microfabrication processes (e.g., lithography, deposition, patterning, and etching) that are costly, time-consuming, and difficult to implement [1,2,3,7]. These challenges can make it difficult to fabricate and assemble sub-mm scale wireless implants, which can limit their use in real-world applications and research.

In this research, we have developed a novel post-CMOS process technique to fabricate the smallest wirelessly powered, microelectronic brain implant that does not require post-CMOS microfabrication techniques. The proposed implant uses a surface-mount device (SMD) inductor to harvest electromagnetic energy, which has not been demonstrated previously to the best of our knowledge. In this manuscript, we focus on the fabrication, assembly, packaging, and wireless powering of the sub-mm scale stimulating IMD. We also include a simple in vivo proof-of-concept to demonstrate the epicortical implant’s ability to stimulate the brain by looking for immediate early gene expression (c-Fos) as a measure of neural activity. Overall, our novel approach allows for the production of small, wireless brain implants that are more cost-effective and easier to fabricate and assemble than traditional methods.

## 2. Materials and Methods

### 2.1. ASIC

**Figure 2:**
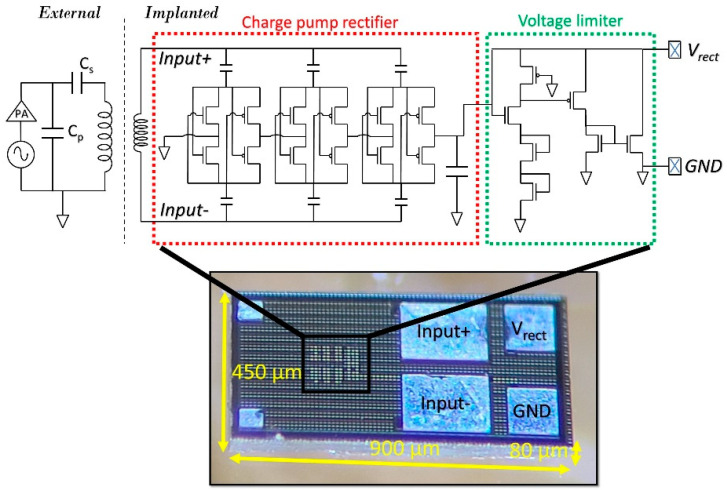
A schematic diagram of the entire system and a micrograph of the ASIC.

The ASIC was designed in X-FAB’s 180 nm silicon-on-insulator (SOI) process and measures 900 × 450 × 80 µm^3^. The circuit diagram is shown in Figure 2. The circuit encircled in red is the charge pump rectifier, which consists of three rectifier stages that are connected in series. The rectifier was a classic CMOS cross-connected bridge made up of LVT transistors. The rectified voltage was kept below 3 V using a voltage limiter, which shunts excess current if the received RF power levels exceed a certain level. This protects the transistors from breakdown and prevents overstimulation of neural tissue. The charge pump rectifier power conversion efficiency (PCE) was maximized for a certain operating frequency (1.17 GHz) and output load (120 nF in series with 10 kΩ). The reason for selecting a high power frequency is discussed in [8]. The SOI technology improved the PCE by eliminating current leakages to the substrate and minimizing eddy currents in the substrate as large amounts of power are transmitted at a short distance from the implant.

### 2.2. Wireless Link

**Figure 3:**
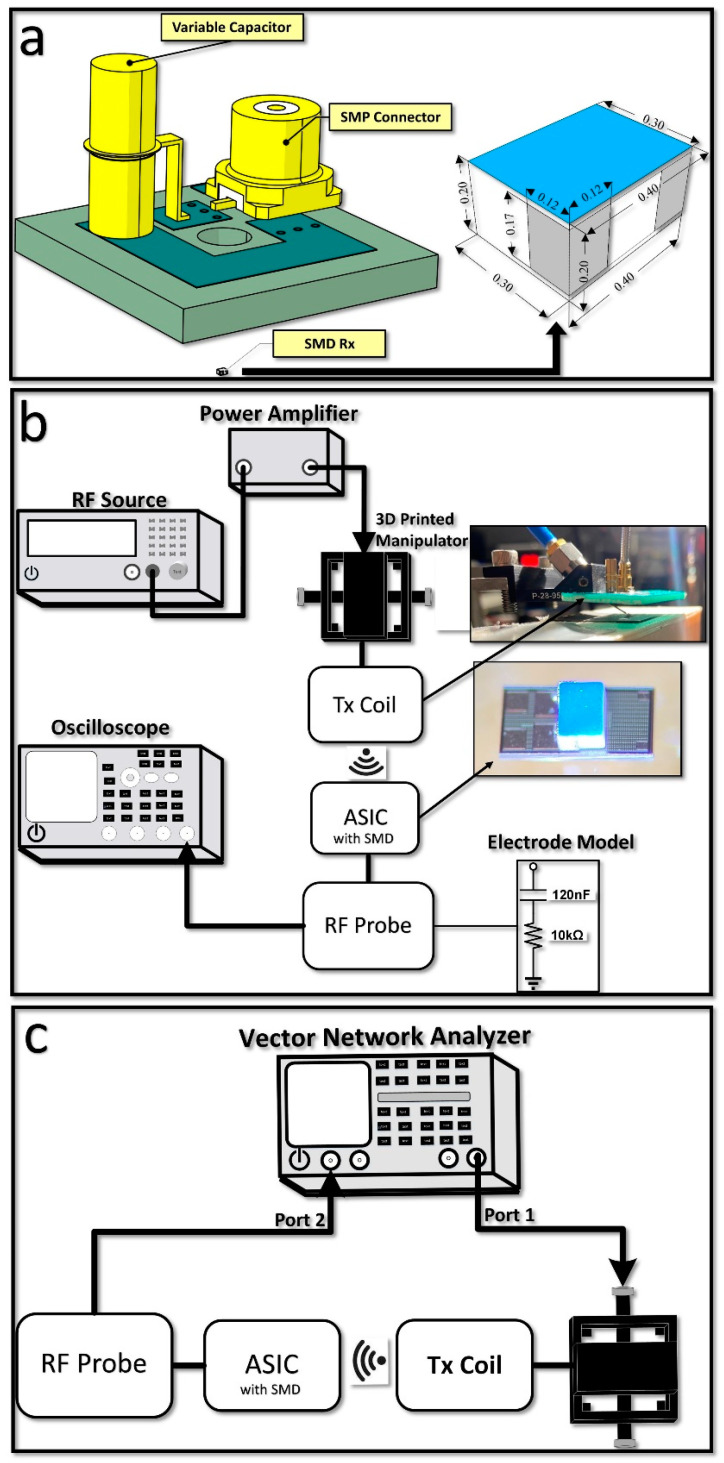
(**a**) HFSS simulation model of the wireless power link, (**b**) drawing/picture of the experimental setup used to measure the rectified voltage, and (**c**) drawing of the experimental setup used to measure the S_12_, S_11_, and S_22_ parameters. For both testbench setups, the Tx–Rx distance was set to 5 mm.

A single-turn transmitter (Tx) coil was built on an FR4 substrate (Figure 3a) based on the optimization methodology described in our past work [5]. The printed circuit board (PCB) also contained the L-match capacitor network, to maximize transmitted power, and an SMP connector. The parallel capacitor was placed at the bottom of the PCB and is not visible in the 3D drawing. The Tx coil had an outer diameter of 10 cm and a trace width of 3 mm. 

As for the ultra-small receiver (Rx) coil, there are three known methods of connecting it to the CMOS ASIC [9]: around CMOS (wire wound around the CMOS chip), in CMOS (fully integrated coils), and above CMOS (on top of CMOS substrate). The in-CMOS coil offers cost effectiveness, smaller volume, and ease of mass production but suffers from poor PTE (primarily due to substrate leakage) [10]. The around-CMOS coil offers a very high-quality factor (Q) but has variability in its electrical properties, making it challenging to resonate at a specific frequency [2]. While the above-CMOS coil combines the advantages of the other two methods, it comes at the cost of a larger volume. Considering these trade-offs, we decided to use the above-CMOS approach for our design.

The Rx is an RF SMD inductor that was obtained from Murata (LQP02HQ20N). It measures 400 × 300 × 200 µm^3^, has an inductance of 20.8 nH, a resistance of 6.5 Ω, a quality factor of 24 at 1.17 GHz, and a self-resonance frequency above 3 GHz. The inductor has a multilayer structure and can, therefore, be considered an air core solenoid. For the surface area, solenoids generally provide higher coupling coefficients than planar coils. Its inductance value was chosen such that the capacitive impedance of the rectifier was eliminated (at 1.17 GHz) once the SMD was connected to the ASIC. The number of turns, materials, and geometry are confidential and thus not provided by Murata. Fortunately, a 3D-encrypted model is accessible and can be used to simulate the wireless link.

Figure 3a shows the Ansys HFSS simulation setup designed to simulate the wireless link. This setup replicates the measurement setup (the one using the vector network analyzer as described below) but omits the RF probe and the ASIC. To account for the impact of the ASIC, the simulated s2p of the Tx–Rx in HFSS and the measured s1p of the ASIC were imported to the Advanced Design System (ADS) software. The Tx PCB includes an SMP connector, two capacitors, and a square-shaped coil. As shown in Figure 3a, the 3D models of the SMP connector and the capacitors were included due to their large size and potential impact on the electromagnetic field in the near field.

Two measurement setups were implemented in this research. These setups did not rely on PCBs or wire bonds to interface with the ASIC. When transmitting large amounts of power at a short distance from the test board, the setup becomes prone to unwanted eddy currents in the PCB traces and wire bonds.

The first setup (Figure 3b) was used to measure the rectified voltage and current passing through the electrode model when the implant was wirelessly powered at a distance of 5 mm. The RF probe (150 µm pitch), which contacted the output bond pads, was connected to an oscilloscope and the electrode equivalent model. The RF signal generator was connected to a power amplifier with a gain of 46 dB at 1.17 GHz. The transmitted power was pulsed (with a 500 µs pulse width) to avoid overheating the Tx coil. Approximately 23 dBm (200 mW) of power was provided to the Tx coil. A 3D-printed plastic manipulator was used to precisely control the distance between the Tx coil and the SMD solenoid and the x-y position. 

The second setup was used to measure the S-parameters of the ASIC in order to calculate the maximum achievable efficiency (PTE_max_) (Figure 3c). The PTE_max_ is defined as the ratio of power delivered to the ASIC to the power amplifier (PA) output power, with the assumption that an ideal matching network is included in the ASIC. In this setup, the RF probe was in contact with the input bond pads of the ASIC. A vector network analyzer (VNA) was connected to the RF probe and Tx coil. To ensure accurate measurements, the parasitic capacitance and inductance caused by the coaxial cable and the probe were removed during the de-embedding calibration procedure (short/open/50 Ω). As in the previous setup, the Tx-–Rx distance was set to 5 mm. It is important to note that the measurements were taken after the die (obtained from the foundry) was further diced to remove the grounded seal edge which significantly impacts the wireless inductive coupling as shown in other works [11]. PTE_max_ under optimal loading conditions is outlined and derived in [12] and is given by:

(1) $${\mathrm{PTE}}_{\max}=\frac{\chi }{(1+{\sqrt{1+\chi )}}^{2}}$$

*χ* can be derived from the *Z*-parameters of the network as:

(2) $$\chi =\frac{{\left|Z_{12}\right|}^{2}}{Re\left(Z_{11}\right)Re\left(Z_{22}\right)-Re{\left(Z_{12}\right)}^{2}}$$

### 2.3. Fabrication and Assembly Process Flow 

**Figure 4:**
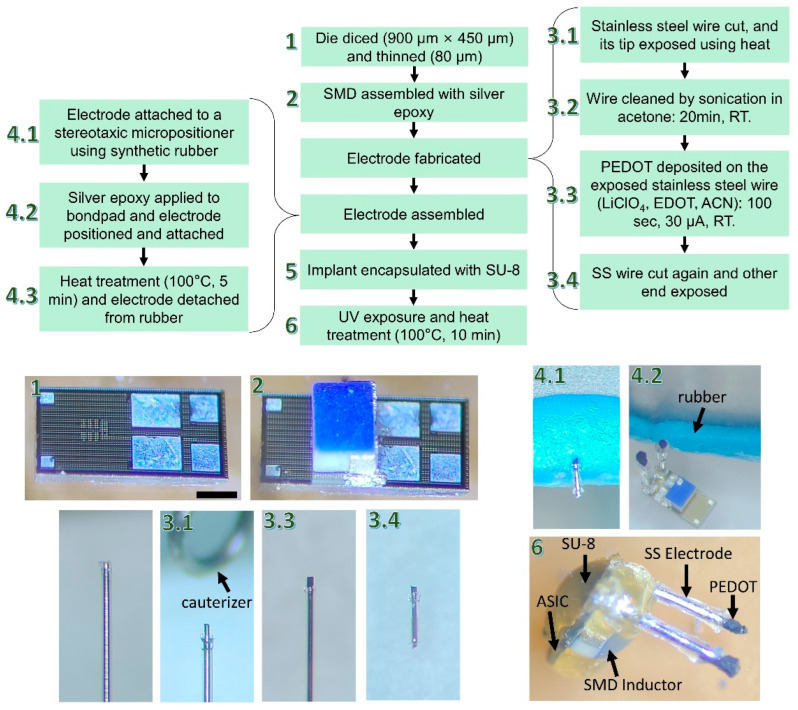
Detailed flowchart of the complete fabrication and assembly process flow of the epicortical IMD and pictures showing some of the steps. The scale bar measures 200 µm.

The entire process flow used to convert the ASIC into a stimulating IMD is shown in Figure 4. The process began by receiving the 3 × 3 mm^2^ dies from the foundry and dicing them down to 900 × 450 µm^2^. The die was then thinned down to 80 µm, after removing approximately 620 µm of silicon (step #1). While it is possible to thin the ASIC down to 30 µm, this would make it more susceptible to microcracks during handling. An optional zincation process can then be carried out to etch the alumina and prevent the Al bond pads from oxidizing. The next step was to attach the SMD solenoid to the ASIC (step #2). This was achieved by adding a small amount of conductive silver epoxy adhesive (8330D, MG Chemicals) on the input bond pads and carefully placing the SMD on top of it. The ASIC was then placed on a hotplate to cure the epoxy for 5 min at 100 °C. Once the electrical connection to the bond pads had been confirmed using the VNA and probe station, the electrodes were fabricated.

To make the intracortical electrodes, PFA-coated stainless steel (SS) wires were purchased from A-M Systems. The SS wire had a diameter of 76.2 µm and a length of approximately 1.5 mm; although, the length can be adjusted depending on the specific layer of the cortex that it needs to be interfaced with. The insulating PFA layer on one end of the electrode was removed to expose the stainless steel (total surface exposed ≈ 0.05 mm^2^) by carefully melting the PFA using a cauterizer (step #3.1). Conducting polymers have often been utilized to improve the performance of metallic electrodes. In this research, PEDOT was used to significantly decrease the impedance of the stimulating electrode pair. The electropolymerization process (carried out galvanostatically) used to deposit a conducting polymer on a metal electrode was inspired by the work of Rossetti et. al. [13]. Before electrodeposition, the electrode was rinsed with 15 min of sonication in acetone and then in deionized water. The polymerization solution consisted of 5 mL of the ACN solvent, 15 µL of the EDOT monomer [30 mM], and 0.06 g of the LiClO4 dopant [120 mM]. Electropolymerization was carried out in a three-electrode electrochemical cell using a galvanostat (Interface 1000, Gamry Instruments, Warminster, PA, USA) with a delivered current of 30 µA for 100 s. Silver/silver chloride (Ag/AgCl) was used as the reference electrode, a Pt wire as the counter electrode, and the SS wire as the working electrode. Once PEDOT deposition was completed, the wire was cut again, and the other end of the electrode was exposed (step #3.4). Connecting the electrodes to the output bond pads (V_rect_ or GND) of the ASIC manually proved to be challenging. To assist with this task, we used a micropositioner on a stereotaxic instrument with rubber to hold the electrode in place. Then, we applied conductive silver epoxy to one of the output bond pads and aligned the electrode with the bond pad. The epoxy was heated to attach the electrode, after which it was detached from the micropositioner. This process was repeated for the second electrode (step #4.2). The implant was then coated with a thick layer (>300 µm) of SU-8 (Kayaku, MA, USA). This was performed by immersing the ASIC in SU-8 while holding onto the electrodes. The SU-8 material was chosen for its chronic biocompatibility as it does not show apparent signs of tissue damage or inflammatory reaction over many months in vivo [14,15]. Finally, SU-8 was heated and exposed to UV until it developed a yellowish color (step #6). 

### 2.4. Electrode Impedance Characterization

To verify that the impedance of the stainless steel electrode coated with PEDOT had decreased, we conducted electrochemical impedance spectroscopy (EIS) measurements using a galvanostat (Interface 1000 from Gamry Instruments, Warminster, PA, USA). The measurements were carried out in a PBS solution, and the frequency range was from 1 Hz to 10 kHz with a 10 mV sinusoidal excitation voltage. The results are shown in the form of Bode plots.

### 2.5. In Vivo Experimental Design

The following proof-of-concept experiment aimed to show that the fabricated wirelessly powered implantable medical device can increase c-Fos levels (an indirect marker of neural activity). All research protocols were approved and monitored by the Massachusetts General Hospital (MGH) Institutional Animal Care and Use Committee (IACUC). One male Sprague Dawley rat (Charles River Labs, MA, USA) was used in the experiment. After shaving the fur over the surgical site, the anesthetized animal was immobilized on a stereotaxic frame, and the scalp was disinfected and numbed with lidocaine. A sagittal incision (up to 1.5 cm in length) was made in the skin over the skull. A craniotomy 4 × 2 mm^2^ was performed to expose the motor cortex in both hemispheres. After removing the dura, the IMD was placed on the surface of the right hemisphere’s cortex to deliver current pulses to the motor cortex (M1), while the left hemisphere’s M1 region served as a control. The Tx coil was positioned 5 mm above the implant and was supplied with power pulses of 35 dBm (~3.2 W) at 1.17 GHz. More power than required was transmitted to account for any possible misalignments. The M1 region was stimulated for 40 min at a frequency of 3 Hz using monophasic current pulses of 500 μs width. The animal was then euthanized with a solution of pentobarbital after 80 min under anesthesia to provide sufficient time for the expression of c-Fos. The rat was perfused with ice-cold 1X PBS with 10 U/mL heparin followed by 4% PFA. The brain was extracted and immersion-fixed in 4% PFA and hand-delivered to LifeCanvas Technologies. The entire rat brain was processed using the SHIELD protocol. The brain was cleared for 7 days and then actively immunolabeled using SmartBatch+ (LifeCanvas Technologies, Cambridge, MA, USA). The sample was stained with primaries (rabbit anti-c-Fos antibody) and incubated for refractive index matching before imaging using a SmartSPIM light sheet microscope.

## 3. Results

### 3.1. Benchtop Measurement Results 

#### 3.1.1. ASIC and Wireless Link Characterization 

**Figure 5:**
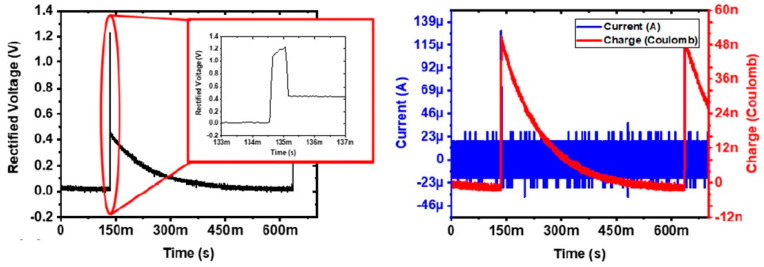
(**Left**) Measured rectified voltage and (**Right**) current through the load when the device is wirelessly powered at a distance of 5 mm and connected to an RC load (representing the microelectrode).

Figure 5 demonstrates that the ASIC can rectify the incoming AC signal to provide a current amplitude of 128 µA to the electrode equivalent model. However, as the current prototype is only capable of generating monophasic stimulus pulses, it takes approximately 450 ms for the rectified voltage to reach 0 V. This means that high-frequency stimulation (>2 Hz) is not possible using the demonstrated device but could easily be enabled if it were to generate biphasic pulses.

**Figure 6:**
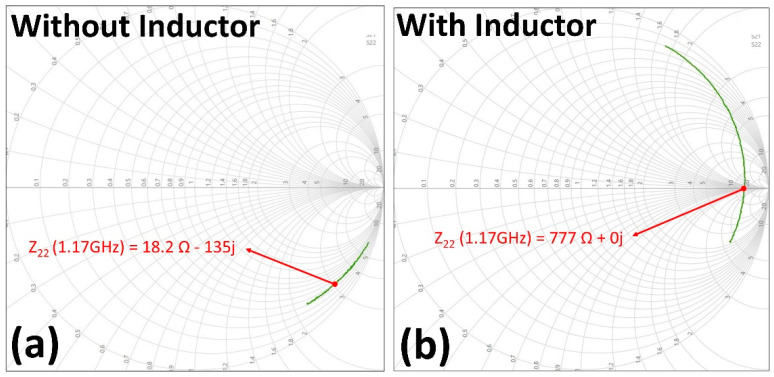
Measured S_22_ parameters plotted on a Smith chart without (**a**) and with (**b**) the SMD inductor. Z_22_ values are displayed for 1.17 GHz.

The Smith chart (Figure 6) shows that resonance was achieved at 1.17 GHz, which is in agreement with the calculated value. It was observed that when the contact resistance between the surface mount device and bond pad was high due to poor bonding, the resonance frequency did not match expectations. 

**Figure 7:**
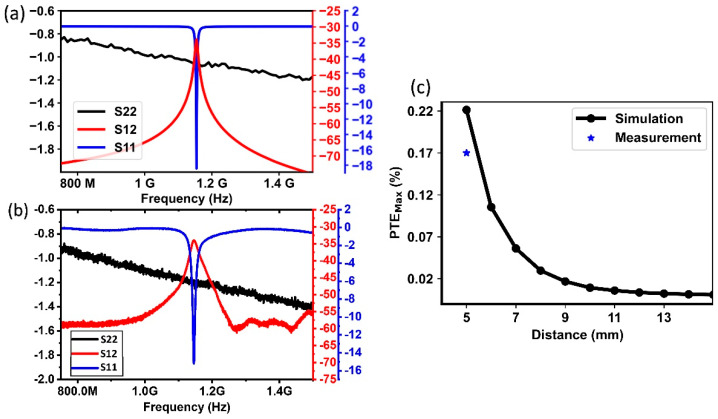
(**a**) Simulated and (**b**) measured S_11_, S_12_, S_22_ parameters of a 2-coil inductive link through air with 5 mm Tx–Rx separation. (**c**) Simulated and measured PTE_max_ as a function of Tx–Rx distance.

The S-parameter results are shown in Figure 7a,b. Simulation results show an S12 of 33.9 dB at 1.15 GHz, while the measurement results show an S12 of 34 dB at 1.17 GHz. The small difference in resonance frequency can be minimized by more precisely tuning the two variable capacitors in HFSS. Measurement results are in very good agreement with simulation results with regard to the S12 at resonance. Outside resonance, the S12 settled around -60 dB for measurements and −75 dB for simulations. The main contributing factor to the mismatch was likely the impact of electromagnetic interference (EMI) on the RF probe and the coax cable. It is worth noting that the peak S12 was about −65 dB when the SMD component was removed, indicating that power harvested from EMI is negligible. It can also be observed that the measured S12 values are below -50 dB for frequencies below 1.125 GHz and frequencies above 1.2, indicating that an implantable medical device can be addressed individually among multiple IMDs with different resonant frequencies. 

The measured PTE_max_ was 0.17% at 1.17 GHz. This value allows us to estimate the power transfer efficiency if a matching network is added to the ASIC. It is important to note that the current prototype does not include a matching network, and the load is not close to its optimal value. The simulated PTE_max_ shows that if the Tx–Rx distance were to be increased above 11 mm, then it would be impractical to continuously power the device. For instance, at a distance of 11 mm or more, if we transmit 1 W of power, we would receive less than 100 µW at the SMD solenoid. Therefore, either the Tx–Rx has to be kept below 10 mm or a pulsed powering scheme needs to be utilized for this sub-mm scale implant. 

#### 3.1.2. Electrode Characterization

**Figure 8:**
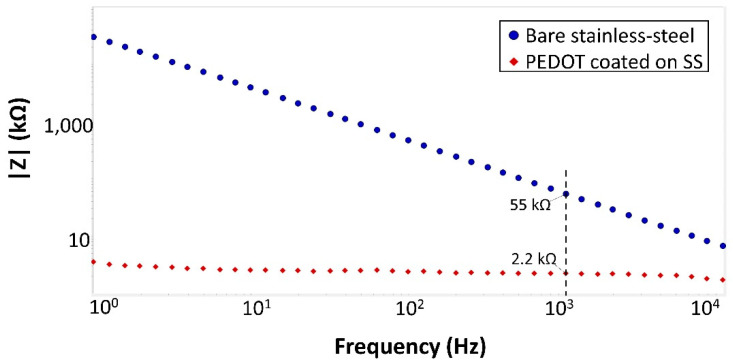
Impedance spectra comparing PEDOT-coated stainless steel electrode with the uncoated stainless steel electrode.

As shown in the EIS data (Figure 8), the impedance of the PEDOT-coated electrode is 25 times smaller than that of the bare stainless steel electrode at 1 kHz. This decrease in impedance is consistent with previous findings in other studies that utilized PEDOT coating on electrodes [16]. We have also assessed the adhesion strength of the electrode–bond pad interface by penetrating a 0.6% gel brain phantom twenty times using the same IMD. Upon examination under a microscope, we observed that there was no visible detachment of the interface. The same IMD was then used for the in vivo experimental validation. This suggests that the adhesion strength of the interface is sufficient for animal experiments with the dura removed.

### 3.2. In Vivo Experimental Results 

**Figure 9:**
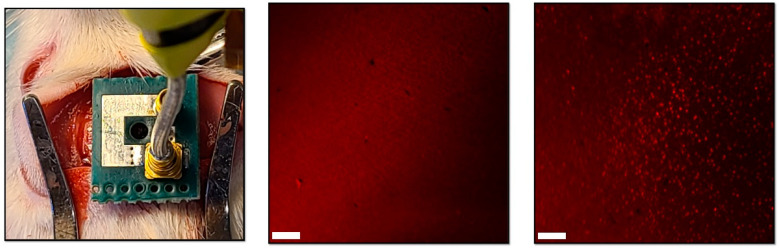
Technology validation using immunofluorescence imaging: Picture of the rat experiment during wireless stimulation. c-Fos expression in the left (control) and right (stimulated) hemispheres of a rat brain. Scale bar is 100 µm.

Figure 9 shows representative immunofluorescence images of the M1 region in the left and right hemispheres. We observed strong c-Fos activation in the right M1 region and none in the left M1 region, indicating that the elevated c-Fos expression was indeed induced by the stimulating IMD.

## 4. Discussion and Conclusions

Single-channel, sub-mm scale, wireless devices have the potential to enable new or improved neural interfacing applications. This work demonstrates a fabrication and assembly method that is simple to replicate, unlike similar-sized implantable medical devices that require complex and costly microfabrication processes. The described fabrication/assembly process flow took less than 2 h to complete at a minimal cost. By assembling the solenoid and electrodes directly to the ASIC, we were able to eliminate the use of PCBs, enabling a significant reduction in the size of the implant. The estimated volume (without the penetrating electrodes) of the IMD is approximately 0.27 mm^3^, making it the smallest wirelessly powered IMD that did not require post-CMOS microfabrication techniques (Table 1). It is worth noting that, although the described fabrication process was used to build a stimulating IMD, the same process can be modified to make recording IMDs by altering the circuit and increasing the impedance of the electrodes (e.g., by reducing the exposed area). Future research directions include repeating the characterization of the wireless link with tissue (scalp/skull/meninges) between the Tx and Rx and exploring distributed neuromodulation in the cortex of animal models.

## Figures and Tables

**Table 1:** Comparison table of the recent epicortical, stimulating, wireless microdevices.

References	[17]	[18]	[14]	This Work
CMOS process	180 nm HV	65 nm	N/A	180 nm SOI
Wireless link	Ultrasound	Ultrasound	2-coil inductive	2-coil inductive
Electrode material	Pt	PEDOT	PtIr	PEDOT
Surface area (mm^2^)	3.3	0.3	0.79	0.05
Impedance (kΩ)	<1	4	-	2.2
Encapsulation material	PDMS	Parylene	SU-8	SU-8
Animal model	Frog sciatic	Rat sciatic	Rat peroneal	Rat brain
Reliance on microfabrication techniques	None	Light	Heavy	None
Volume (mm^3^)	39	1.7	1.39	0.27

## Data Availability

The data presented in this study are available on request from the corresponding author.

## References

[1] Lee J., Leung V., Lee A.H., Huang J., Asbeck P., Mercier P.P., Shellhammer S., Larson L., Laiwalla F., Nurmikko A. (2021). Neural recording and stimulation using wireless networks of microimplants. Nat. Electron..

[2] Yeon P., Mirbozorgi S.A., Ash B., Eckhardt H., Ghovanloo M. (2016). Fabrication and microassembly of a mm-sized floating probe for a distributedwireless neural interface. Micromachines.

[3] Lee S., Cortese A.J., Mok A., Wu C., Wang T., Park J.U., Smart C., Ghajari S., Khilwani D., Sadeghi S. (2020). Fabrication of Injectable Micro-Scale Opto-Electronically Transduced Electrodes (MOTEs) for Physiological Monitoring. J. Microelectromechanical Syst..

[4] Johnson B.C., Shen K., Piech D., Ghanbari M.M., Li K.Y., Neely R., Carmena J.M., Maharbiz M.M., Muller R. timDust: A 6.5mm3, wireless ultrasonic peripheral nerve stimulator with 82% peak chip efficiency. Proceedings of the 2018 IEEE Custom Integrated Circuits Conference (CICC).

[5] Khalifa A., Liu Y., Karimi Y., Wang Q., Eisape A., Stanaćević M., Thakor N., Bao Z., Etienne-Cummings R. (2019). The Microbead: A 0.009 mm^3^ Implantable Wireless Neural Stimulator. IEEE Trans. Biomed. Circuits Syst..

[6] Khalifa A., Lee S., Molnar A.C., Cash S. (2021). Injectable wireless microdevices: Challenges and opportunities. Bioelectron. Med..

[7] Lee A.H., Lee J., Laiwalla F., Leung V., Huang J., Nurmikko A., Song Y.K. (2020). A scalable and low stress post-cmos processing technique for implantable microsensors. Micromachines.

[8] Khalifa A., Zhang J., Leistner M., Etienne-Cummings R. A compact, low-power, fully analog implantable microstimulator. Proceedings of the IEEE International Symposium on Circuits and Systems (ISCAS).

[9] Feng P., Yeon P., Cheng Y., Ghovanloo M., Constandinou T.G. (2018). Chip-Scale Coils for Millimeter-Sized Bio-Implants. IEEE Trans. Biomed. Circuits Syst..

[10] Khalifa A., Nasrollahpour M., Sun N., Zaeimbashi M., Chen H., Liang X., Alemohammad M., Etienne-Cummings R., Sun N.X., Cash S. Magnetoelectric versus inductive power delivery for sub-mm receivers. Proceedings of the IEEE Wireless Power Transfer Conference (WPTC).

[11] Khalifa A., Karimi Y., Huang Y., Stanacevic M., Etienne-Cummings R. The Challenges of Designing an Inductively Coupled Power Link for μm-sized On-Chip Coils. Proceedings of the 2018 IEEE Biomedical Circuits and Systems Conference (BioCAS).

[12] Zargham M., Gulak P.G. (2012). Maximum achievable efficiency in near-field coupled power-transfer systems. IEEE Trans. Biomed. Circuits Syst..

[13] Rossetti N., Luthra P., Hagler J.E., Jae Lee A.H., Bodart C., Li X., Ducharme G., Soavi F., Amilhon B., Cicoira F. (2019). Poly(3,4-ethylenedioxythiophene) (PEDOT) Coatings for High-Quality Electromyography Recording. ACS Appl. Bio Mater..

[14] Cho S.-H., Xue N., Cauller L., Rosellini W., Lee J.-B. (2013). A SU-8-based fully integrated biocompatible inductively powered wireless neurostimulator. J. Microelectromechanical Syst..

[15] Márton G., Tóth E.Z., Wittner L., Fiáth R., Pinke D., Orbán G., Meszéna D., Pál I., Győri E.L., Bereczki Z. (2020). The neural tissue around SU-8 implants: A quantitative in vivo biocompatibility study. Mater. Sci. Eng. C.

[16] Cui X., Martin D.C. (2003). Electrochemical deposition and characterization of poly(3,4-ethylenedioxythiophene) on neural microelectrode arrays. Sens. Actuators B Chem..

[17] Charthad J., Chang T.C., Liu Z., Sawaby A., Weber M.J., Baker S., Gore F., Felt S.A., Arbabian A. (2018). A mm-Sized wireless implantable device for electrical stimulation of peripheral nerves. IEEE Trans. Biomed. Circuits Syst..

[18] Piech D.K., Johnson B.C., Shen K., Ghanbari M.M., Li K.Y., Neely R.M., Kay J.E., Carmena J.M., Maharbiz M.M., Muller R. (2020). A wireless millimetre-scale implantable neural stimulator with ultrasonically powered bidirectional communication. Nat. Biomed. Eng..

